# Supplementary material for: Risk of Occupational Latent Tuberculosis Infection among Health Personnel Measured by Interferon-Gamma Release Assays in Low Incidence Countries—A Systematic Review and Meta-Analysis
Source: Int J Environ Res Public Health. 2020 Jan 16;17(2):581. doi: 10.3390/ijerph17020581 (PMC7027002; doi:10.3390/ijerph17020581)
Supplement: Supplementary file 1 [file ijerph-17-00581-s001.pdf]

Table S1. Eligibility criteria for the inclusion of studies.

Inclusion	Exclusion
Population	
• Healthcare workers (HCWs) whose occupations meant they had either direct contact with patients (doctors, nursing staff and assistants, students, various therapists) or indirect or no contact with patients, but were exposed to infected material or an infected environment (laboratory workers, cleaning staff, administrative employees)<br>• HCWs from low-incidence countries (≤ 40 cases per 100,000 population)	• Work outside healthcare institutions<br>• HCWs from high-incidence countries (> 40 cases per 100,000 population)<br>• Selective occupational groups (e.g. students or professionals from high-incidence countries)
Exposure	
• Occupational exposure, transmission, occupational disease<br>• Direct and indirect exposure (see above)<br>• Use of immunological tests only, such as IGRAs, to diagnose LTBI:<br>o QuantiFERON® test (QFT),<br>o T-SPOT.TB test<br>o Enzyme-linked immunospot (ELISpot)<br>• Routine TB screenings in connection with occupational health check-ups	• Exposure and diagnosis in a non-occupational context and outside healthcare institutions<br>• Environmental analyses following a TB outbreak
Outcome	
• LTBI diagnosed using immune-based testing<br>• Findings differentiated by occupational group, job category or age	• LTBI only diagnosed using the tuberculin skin test (TST)<br>• IGRA only used to confirm positive TSTs[1]<br>• Disclosure of conversion and reversion rates alone
Study design	
• Peer-reviewed publications<br>• Cohort, case-control and cross-sectional studies<br>• Studies from which concrete data on the prevalence and incidence of LTBI can be extracted and calculated	• Lead articles and letters<br>• Comments<br>• Conference contributions<br>• Policy statements or reports<br>• Case reports
Publication period	
• Studies published between 2005 (market launch of IGRA) and 31/01/2019. Search update on 15/08/2019	
Languages	
• English, German, Dutch, French, Italian, Portuguese, Spanish, Turkish	• Languages not – or insufficiently – spoken by the study group

[1] Studies which used TSTs to diagnose LTBI were only included if IGRA tests were used simultaneously and the results were presented separately.

Table S2. Keywords included in the search strategy for all databases.

Population	Exposure (Measurement)	Outcome
Health personnel[Mesh]	Disease Transmission, Infectious[Mesh]	Tuberculosis[Mesh]
Health care worker*	Interferon-gamma Release Tests [Mesh]	TB infection
Health care staff*	IGRA	Latent tuberculos*
Health professional*	Quantiferon*	TB
Hospital staff*	QFT	LTBI
Hospital personnel	T-SPOT	
Nurs*		
Students, Health Occupations[Mesh]		
Nursing student*		
Nursing staff*		
Medical staff*		
Laboratory personnel[Mesh]		
Physician*		
Dentist*		
Caregiver*		

Table S3. PubMed specific search strategy.

Category	Keywords [tw] and MeSH headings
Outcome	((((("Tuberculosis"[Mesh]) **OR** Tuberculos*[tw]) **OR** TB infection*[tw]) **OR** Latent tuberculos*[tw]) **OR** TB[tw]) **OR** LTBI[tw]
	**AND**
Exposition	(((((("Interferon-gamma Release Tests"[Mesh]) **OR** Interferon-gamma release assay*[tw]) **OR** Quantiferon*[tw]) **OR** QFT[tw]) **OR** IGRA[tw]) **OR** T-SPOT*[tw]) **OR** "Disease Transmission, Infectious"[Mesh]
	**AND**
Population	((((((((((((((((("Health Personnel"[Mesh]) **OR** health care worker*[tw]) **OR** healthcare worker*[tw]) **OR** health care staff*[tw]) **OR** healthcare staff*[tw]) **OR** health professional*[tw]) **OR** hospital staff*[tw]) **OR** health personnel[tw]) **OR** nurs*[tw]) **OR** "Students, Health Occupations"[Mesh]) **OR** nursing student*[tw]) OR nursing staff*[tw]) **OR** medical staff*[tw]) **OR** "Laboratory Personnel"[Mesh]) OR laboratory personnel[tw]) **OR** physician*[tw]) **OR** dentist*[tw]) OR caregiver*[tw]) **OR** hospital personnel[tw]

Study or Subgroup	Nurses Events	Nurses Total	Controls Events	Controls Total	Odds Ratio M-H, Random, 95% CI
Adachi 2013	6	72	12	93	0.61 [0.22, 1.72]
Al Hajoj 2016	204	849	195	746	0.89 [0.71, 1.12]
Alvarez-Leon 2009	2	18	6	116	2.29 [0.43, 12.35]
Babayigit 2014	7	33	12	63	1.14 [0.40, 3.25]
Bukhary 2018	12	160	44	360	0.58 [0.30, 1.14]
El-Helaly 2014	149	547	184	865	1.39 [1.08, 1.78]
El-Sokkary 2015	34	116	4	16	1.24 [0.37, 4.13]
Girardi 2009	16	67	13	48	0.84 [0.36, 1.97]
Gran 2013	5	222	13	165	0.27 [0.09, 0.77]
Guanche Garcell 2014	3	128	3	74	0.57 [0.11, 2.89]
Harada 2006	18	167	15	165	1.21 [0.59, 2.49]
Kargi 2017	6	25	17	75	1.08 [0.37, 3.13]
Larcher 2012	39	242	76	379	0.77 [0.50, 1.17]
Moucaut 2013	48	217	93	417	0.99 [0.67, 1.47]
Schablon 2014	158	1962	160	1861	0.93 [0.74, 1.17]
Talebi-Taher 2011	10	105	7	95	1.32 [0.48, 3.63]
Tanabe 2017	15	348	18	306	0.72 [0.36, 1.46]
Targowski 2014	10	116	17	189	0.95 [0.42, 2.16]
Torres Costa 2011	317	1204	636	1680	0.59 [0.50, 0.69]
Uto 2014	13	479	15	472	0.85 [0.40, 1.81]
**Total (95% CI)**		**7077**		**8185**	**0.87 [0.72, 1.05]**
Total events	1072		1540		

Heterogeneity: Tau² = 0.07; Chi² = 46.81, df = 19 (P = 0.0004); I² = 59%
Test for overall effect: Z = 1.48 (P = 0.14)

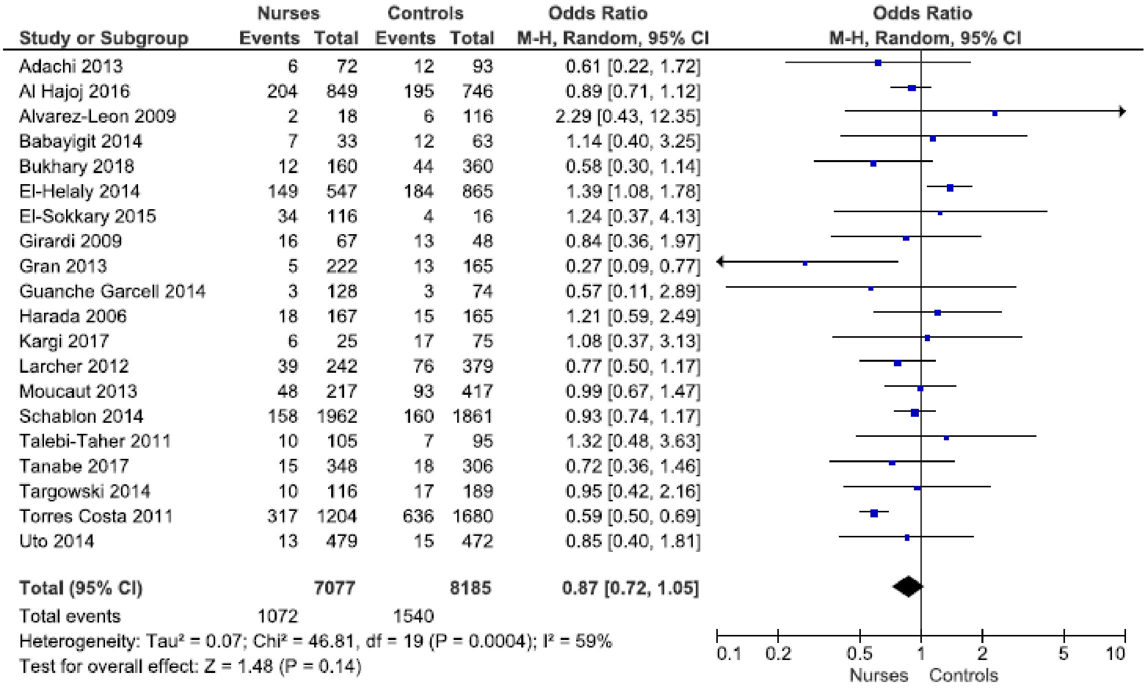

Figure S1. Forest plot of the LTBI prevalence in nurses by IGRA in low incidence countries.

Study or Subgroup	Physicians Events	Physicians Total	Controls Events	Controls Total	Odds Ratio M-H, Random, 95% CI
Adachi 2013	5	53	13	112	0.79 [0.27, 2.35]
Al Hajoj 2016	9	44	390	1551	0.77 [0.36, 1.61]
Alvarez-Leon 2009	1	15	7	119	1.14 [0.13, 9.99]
Babayigit 2014	7	34	12	62	1.08 [0.38, 3.07]
Bukhary 2018	20	100	36	420	2.67 [1.47, 4.85]
El-Helaly 2014	28	197	305	1215	0.49 [0.32, 0.75]
Girardi 2009	1	18	28	97	0.14 [0.02, 1.14]
Gran 2013	6	60	12	327	2.92 [1.05, 8.10]
Guanche Garcell 2014	2	42	4	160	1.95 [0.34, 11.03]
Harada 2006	1	29	32	303	0.30 [0.04, 2.30]
Kargi 2017	11	48	12	52	0.99 [0.39, 2.52]
Larcher 2012	27	112	88	509	1.52 [0.93, 2.48]
Moucaut 2013	7	59	134	575	0.44 [0.20, 1.00]
Schablon 2014	45	583	273	3240	0.91 [0.65, 1.26]
Talebi-Taher 2011	2	54	15	146	0.34 [0.07, 1.52]
Tanabe 2017	17	210	16	444	2.36 [1.17, 4.76]
Targowski 2014	3	52	24	253	0.58 [0.17, 2.02]
Torres Costa 2011	246	635	707	2249	1.38 [1.15, 1.66]
Uto 2014	4	147	24	804	0.91 [0.31, 2.66]
**Total (95% CI)**		**2492**		**12638**	**1.01 [0.76, 1.36]**
Total events	442		2132		

Heterogeneity: Tau² = 0.20; Chi² = 54.76, df = 18 (P < 0.0001); I² = 67%
Test for overall effect: Z = 0.10 (P = 0.92)

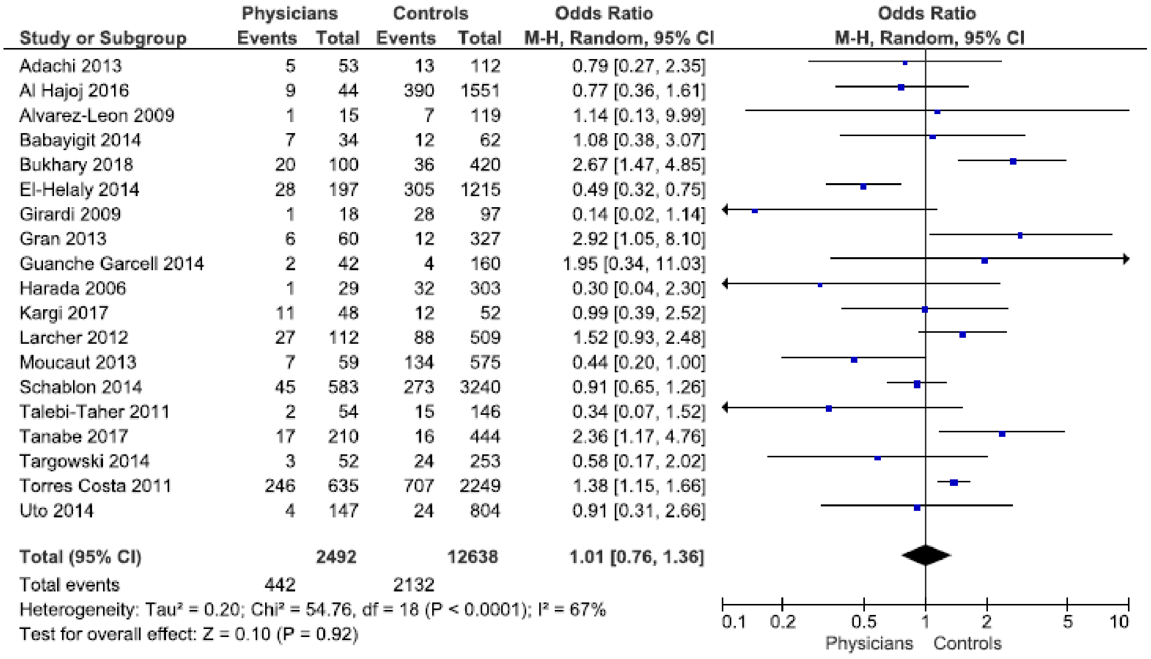

Figure S2. Forest plot of the LTBI prevalence in physicians by IGRA in low incidence countries.

Study or Subgroup	Lab workers Events	Lab workers Total	Controls Events	Controls Total	Odds Ratio M-H, Random, 95% CI
Adachi 2013	3	12	15	153	3.07 [0.75, 12.57]
Babayigit 2014	1	17	18	79	0.21 [0.03, 1.71]
Bukhary 2018	4	50	52	470	0.70 [0.24, 2.02]
El-Sokkary 2015	4	16	34	116	0.80 [0.24, 2.67]
Gran 2013	5	99	13	288	1.13 [0.39, 3.24]
Harada 2006	4	21	29	311	2.29 [0.72, 7.26]
Kargi 2017	3	20	20	80	0.53 [0.14, 2.00]
**Total (95% CI)**		**235**		**1497**	**1.01 [0.58, 1.77]**
Total events	24		181		

Heterogeneity: Tau² = 0.16; Chi² = 8.29, df = 6 (P = 0.22); I² = 28%
Test for overall effect: Z = 0.04 (P = 0.97)

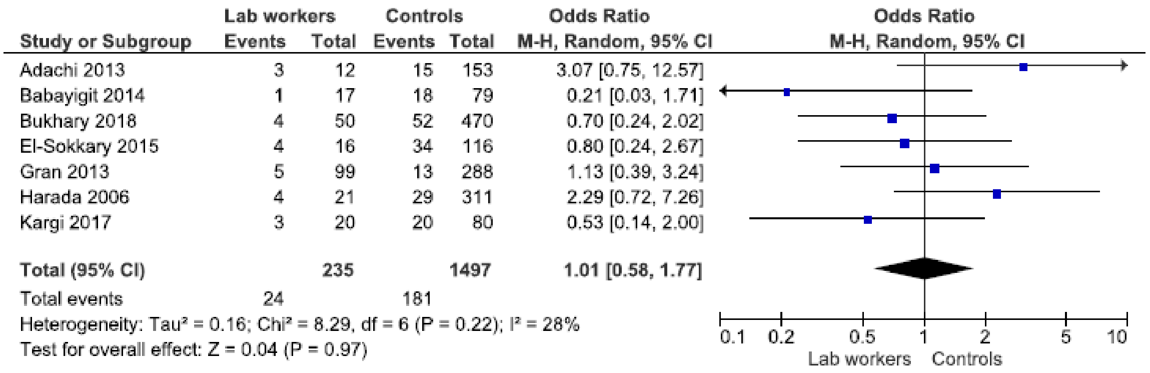

Figure S3. Forest plot of the LTBI prevalence in laboratory workers by IGRA in low incidence countries.
